# Daily Ageist Attitudes, Subjective Age, and Memory Failures: A Multi-Country Integrated Data Analysis

**DOI:** 10.1093/geroni/igaf122.1844

**Published:** 2025-12-31

**Authors:** Shevaun Neupert, Reyyan Can, Shalini Mukherjee, Jana Nikitin, Oksana Senyk, Christiane Hoppmann, Kvitoslava Khorob, Minxia Luo

**Affiliations:** North Carolina State University, Raleigh, North Carolina, United States; North Carolina State University, Raleigh, North Carolina, United States; North Carolina State University, Raleigh, North Carolina, United States; University of Vienna, Vienna, Wien, Austria; WSB Merito University in Gdansk, Gdansk, Pomorskie, Poland; University of British Columbia, Vancouver, British Columbia, Canada; Ukrainian Catholic University, Lviv, L’vivs’ka Oblast’, Ukraine; University of Zurich, Zürich, Switzerland

## Abstract

Ageist attitudes reflect negative views about others’ aging and are negatively associated with health and well-being. However, the ways in which ageist attitudes may fluctuate on a daily basis, and how those fluctuations are coupled with everyday memory functioning, are underexplored. Applying a cross-cultural perspective and leveraging 14-day daily diary data from the USA, Switzerland, Türkiye, Germany, India, Ukraine, Israel, Austria, Czech Republic, and Canada, the goal of the current study was to identify within-person fluctuations in daily ageist attitudes and connect those fluctuations to everyday memory functioning. Multilevel models were conducted across a mixture of integrated data analysis (for data that could be shared) and coordinated data analysis (standardized models applied to locally controlled data) to maximize the inclusion of countries. Estimates of within-person variability in daily ageist attitudes ranged from 13% (Germany) to 44% (India). Conditional models indicated varying patterns across countries. For example, in the US and Germany, daily increases in ageist attitudes were associated with increases in memory failures (reflecting lower levels of everyday memory functioning). However, in Canada and Türkiye, daily ageist attitudes were not associated with memory failures. The current results suggest that ageist attitudes move to varying degrees on a daily basis, and that their importance for everyday memory functioning may depend on cultural context.

